# PrESOgenesis: A two-layer multi-label predictor for identifying fertility-related proteins using support vector machine and pseudo amino acid composition approach

**DOI:** 10.1038/s41598-018-27338-9

**Published:** 2018-06-13

**Authors:** Mohammad Reza Bakhtiarizadeh, Maryam Rahimi, Abdollah Mohammadi-Sangcheshmeh, Vahid Shariati J, Seyed Alireza Salami

**Affiliations:** 1 Department of Animal and Poultry Science, College of Aburaihan, University of Tehran, Tehran, Iran; 2 Genome Center, National Institute of Genetic Engineering and Biotechnology, Tehran, Iran; 3 University of Tehran, Tehran, Iran

## Abstract

Successful spermatogenesis and oogenesis are the two genetically independent processes preceding embryo development. To date, several fertility-related proteins have been described in mammalian species. Nevertheless, further studies are required to discover more proteins associated with the development of germ cells and embryogenesis in order to shed more light on the processes. This work builds on our previous software (OOgenesis_Pred), mainly focusing on algorithms beyond what was previously done, in particular new fertility-related proteins and their classes (embryogenesis, spermatogenesis and oogenesis) based on the support vector machine according to the concept of Chou’s pseudo-amino acid composition features. The results of five-fold cross validation, as well as the independent test demonstrated that this method is capable of predicting the fertility-related proteins and their classes with accuracy of more than 80%. Moreover, by using feature selection methods, important properties of fertility-related proteins were identified that allowed for their accurate classification. Based on the proposed method, a two-layer classifier software, named as “PrESOgenesis” (https://github.com/mrb20045/PrESOgenesis) was developed. The tool identified a query sequence (protein or transcript) as fertility or non-fertility-related protein at the first layer and then classified the predicted fertility-related protein into different classes of embryogenesis, spermatogenesis or oogenesis at the second layer.

## Introduction

Proteins are involved in different aspects of life activities and play critical roles in various biological processes such as the early stages of life development^1^. Germline developmental events including spermatogenesis and oogenesis, and also other variety of differentiation processes such as embryogenesis and organogenesis are regulated by a number of protein signaling cascades which are critical for normal development^2–5^. Gametogenesis is the first stage in sexual reproduction, by which haploid sperm and egg cells are formed from the diploid gamete cells in the ovaries and testes. This process is called oogenesis in the female and spermatogenesis in the male^2–5^. Embryogenesis (or embryo development) is the development of a fertilized egg that fuses with a sperm, forming a zygote. After zygote stage, many changes occur and the embryo undergoes several mitotic divisions to generate tissues layers that eventually develop into specific organs^6,7^. During oogenesis, spermatogenesis and embryogenesis cells initially proliferate and then differentiate into specific tissues. Moreover, oogenesis and spermatogenesis are tightly regulated complex processes critical for fertility^8,9^. Therefore, because of the importance of proteins related to the fertility, their large-scale identification will provide a knowledge base for detailed understanding of biological processes and the mechanisms underlying each step of spermatogenesis, oogenesis and embryogenesis.

A survey of the UniProtKB/TrEMBL databases showed that a large number of un-reviewed proteins exists, which are not annotated and yet to be reviewed. Furthermore, owing to the availability of large number of proteins generated in postgenomic age, wide varieties of unannotated data sets are accumulated in various species and databases^10^. On the other hand, an investigation of protein folding, structure, and function has remained experimentally costly, time consuming and requires sophisticated technical equipment. Hence, there seems to be some benefit in developing efficient computational approaches that can predict protein functions timely and precisely^8,11–16^. By applying such computational models, it is possible to provide an advantageous and powerful substitutional strategy for automating whole proteome annotation without costly and time-consuming experiments.

Over the years, different methods have been proposed for predicting the putative function of unannotated proteins^17,18^. The sequence similarity-based search tools, such as BLAST and PSI-BLAST are among the most robust approaches that have been extensively applied for predicting the unknown protein annotation^13,19–21^. These approaches become more challenging, once the similarity between the input and target sequences is not too much^19,21–23^. To overcome this obstacle, a great deal of attention has been given recently to predict the protein function by applying machine learning based methods. The reliability and efficiency of such methods are well demonstrated in different areas^12,13,24–27^. The higher performance of these methods can be attributed to their ability to learn the underlying rules in training datasets by optimizing the related parameters during the model development. Among the variety of machine learning algorithms which have been proposed in the literature, support vector machine (SVM) is one of the state-of-the-art algorithms and well suited. It is widely believed that SVM is a most promising classifier in different disciplines because of its high accuracy, as well as its power of high dimensional data handling^12,13,28–32^.

In a previous study^12^, for the first time, a model for identifying proteins related to oogenesis was constructed using SMV. Based on the constructed model, OOgenesis_Pred software was developed, which provides a convenient way to annotate the candidate proteins. For the development of this software, a new algorithm was offered to predict not only the proteins involved in oogenesis, but also those implementing spermatogenesis and embryogenesis processes. It is believed that discrimination of biological functions will become more accurate if a collective approach which considers the different kinds of fertility related proteins and their functions are used. Actually, this kind of multi-prediction systems may lead to deeper informative data. Thus, herein, this study aimed to employ the multi label theory in order to develop a new algorithm based on previous SVM classifier along with informative protein physicochemical features. It is expected that this software will be useful in simultaneously predicting the proteins involved in oogenesis, spermatogenesis and embryogenesis processes. Evaluation through a five-fold cross validation and independent test dataset were applied to prove the validity of this method and to check its efficiency, reliability and robustness for prediction of fertility-related proteins.

## Methods

### Datasets

To develop a powerful statistical predictor tool and to train and test it, a high quality and objective benchmark dataset is need. This step is the most important concern in any machine learning method^33,34^. To this end, the following steps were performed:

1. The proteins sequences were collected through searching the UniProtKB database (release 2017_4) with gene ontology terms “oogenesis”, “spermatogenesis” and “embryogenesis”, individually, and then, the initial positive datasets for each fertility-related protein classes were created.
2. Then, only the reviewed proteins which have been experimentally annotated, with the length <6000 or >60 amino acids were selected.
3. The homologous sequences from the datasets using CD-HIT software^35^ were eliminated to ensure that any two sequences shared a pairwise sequence identity of less than 50%.
4. Thereafter, the protein sequences with non-canonical amino acids such as B, X, and Z were excluded.

In this study and by adopting the aforementioned steps, a total of 345, 641, and 831 proteins for “oogenesis”, “spermatogenesis” and “embryogenesis” classes, respectively were obtained, which constituted the positive datasets in this direction. The protein sequences of the negative dataset were also constructed using the UniProtKB database (release 2017_4)^12,13^. Briefly, the database was depleted by comprehensive searching of all keywords suspicious of implying fertility functionality. Only reviewed proteins with length of <6000 and >60 and canonical amino acids were retained. The CD-HIT software with a 50% cutoff was used to remove the highly similar sequences.

In the next step, the domains of all proteins (negative and positive datasets) were compared with each other and proteins with a shared domain were removed from negative datasets. To accomplish this, all related domains were extracted through Pfam database. This enabled the construction of a more reliable negative dataset, which is solely constructed by non-fertility related proteins. Eventually, a number of 342,891 non-fertility related protein sequences were obtained as negative dataset.

The resulting positive (minority class) and negative (majority class) datasets were extremely unbalanced, an issue which is known as the class imbalance problem. Such an imbalance always has undeniable impact on classification results and would lead to a higher prediction rate in favor of majority class^36^. To overcome this issue, a commonly used approach, called random sampling solution (reducing the majority class)^37^, was used. For this purpose, proteins were randomly selected from the negative dataset at the same size of the positive datasets (without replacement). This was performed to generate a balanced benchmark dataset and to minimize influences of the larger negative dataset. However, the efficiency and capability of the predictor cannot be accessed using only one random sample from the negative dataset^38^. Hence, to increase the confidence which is preserved by the present diversity in the negative dataset in random sampling processes, each positive dataset was mixed with five non-overlapping negative samples (drawn without replacement from negative dataset). In other words, the negative dataset was first divided into five sub-datasets with non-overlapping sequences, where the number of sequences in each sub-dataset was equal to that of the positive dataset. Then, the negative sub-datasets and the positive dataset were combined to form a new dataset. Eventually, five new datasets were constructed for each class. Moreover, to create a general fertility-related protein class, all the positive datasets were incorporated and mixed with five different negative sub-datasets. By so doing, 20 benchmark datasets were constructed, with each being applied to train a different SVM model (three classes including embryogenesis, spermatogenesis and oogenesis along with incorporation of all these classes, and each class was combined with five different negative samples).

**Figure 1:**
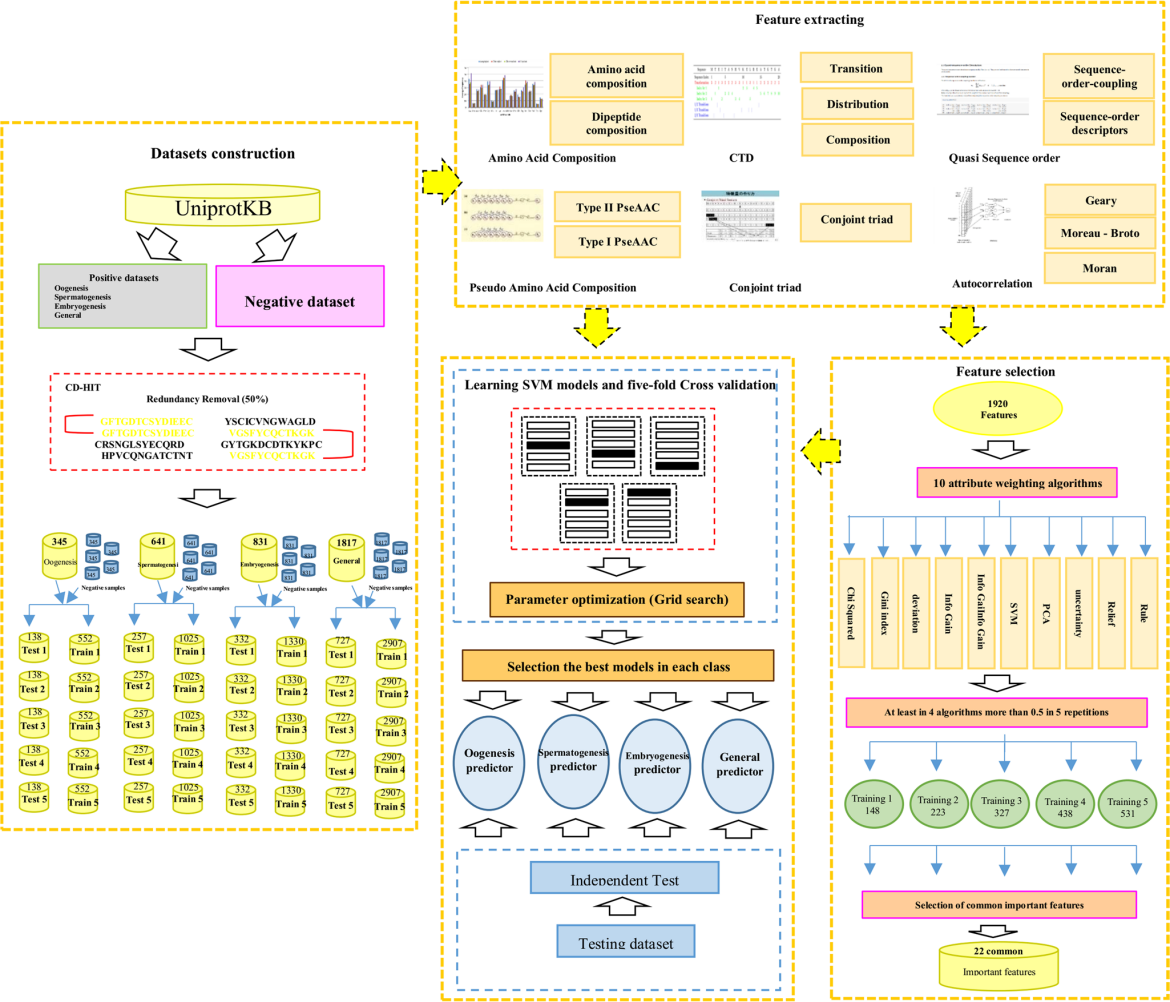
Summary of our pipeline for developing PrESOgenesis.

It is worth noting that cd-hit-2d tool from CD-HIT package was applied across the positive and negative sequences in each benchmark dataset to remove the sequences with 100% identity. This task was possible due to the incomplete annotation of protein sequences in UniProtKB database. Therefore, it is not too far from expected that some negative sequences are identical with positive sequences in each benchmark dataset. The complete information and details of these benchmark datasets is provided in Supplementary File 1. Also, the exact number of proteins in each of the train and test datasets are displayed in Fig. 1.

### Extraction of protein features

Owing to all the existing machine-learning methods which can only handle vector but not sequence samples, a crucial step in computational biology and in the construction of machine learning-based classifier is the formulation of protein sequences with an effective mathematical vector or a discrete model. In this regard, the pseudo amino acid composition (PseAAC), proposed by Chou (Chou’s PseAAC)^39^, is an efficient and widely used method for the conversion of a protein sequence to a vector for developing different predictors^12,13,33,40–48^. The PseAAC makes use of a set of more than 20 discrete factors to represent a protein sequence and captures its key features without completely losing its sequence-pattern information^19^. In the current study, using the concept of Chou’s PseAAC, the protein sequences were encoded with a multi-feature integration strategy to fuse the six different modes of Chou’s PseAAC, using protr package. This package offers a comprehensive and unique tool for generating diverse sequence descriptors of protein sequences^49^. In total, a number of 1920 features were extracted including a 420 dimensional vector indicating amino acid composition and dipeptide composition, a 720 dimensional vector representing Moreau-Broto autocorrelation, Moran autocorrelation and Geary autocorrelation descriptors, a 147 dimensional vector indicating composition, distribution and transition, a 160 dimensional vector indicating sequence-order-coupling number and quasi-sequence-order descriptors, a 130 dimensional vector indicating Pseudo amino acid composition (Type I PseAAC) and amphiphilic pseudo amino acid composition (Type II PseAAC), and a 343 dimensional vector indicating Conjoint Triad descriptors. Previous studies have shown the efficiency and robustness of these features in predicting the protein function^12,13^. The detailed description of these features is provided in Supplementary File 2 (S1).

### Support vector machine (SVM)

Different methods have been used to predict the protein function, such as decision tree^50^, random forest^51^, neural network^13^ and ensemble learning^52^. In this study, SVM was applied to develop all possible models for prediction of fertility-related protein, due to its excellent learning ability and good capability for non-linear classification^46,53^. SVM is a supervised learning hypothesis which can transform the non-linearly separable input vector into a high-dimensional Hilbert space and construct an optimal hyperplane to classify two types of samples^54^. As SVM can transform the input vector from a low dimensional space to a higher dimensional space, its generalization power is better than other machine learning methods for majority of classification tasks. Accordingly, this method has been extensively used in bioinformatics for pattern recognition as well as protein structure and function classification^12,13,31,46,55–61^. LIBSVM package (version 3.22)^62^ was used to implement SVM using radial basis function (RBF) as kernel function. The kernel function determines the learning ability of SVM and prediction performance can be improved by an appropriate choice of kernel function^63^. In this study, RBF was used due to its suitability for non-linear classification as well as its good general performance. For achieving the best model, the optimal values of tunable parameter C and the kernel width parameter γ were determined by the grid search method; this method selects the values of parameters with the consideration of the highest accuracy based on five-fold cross validation. To train and test the model, the benchmark datasets were divided into two independent subsets, training (80% of the benchmark dataset) and testing datasets (20% of the benchmark dataset). To carry out five-fold cross-validation, the training dataset was randomly split into five sub-datasets with approximately equal size. For each cross validation, SVM model was trained based on four sub-datasets (called sub-training set) and the other sub-dataset was used as testing set (called sub-testing set). The process was repeated five times to ensure that each sub-dataset was used once as the sub-testing set. The five validation results were then combined to generate a single estimation. Since possibly in the binary classification mode, the constructed model can be overfitted to the training dataset, the testing dataset which is definitely blind to the process of model training, was used to further evaluate the effectiveness of the predictor.

In this study, a novel two-layer classification framework was developed, as the SVM model in the first-layer classifier was trained with all the training datasets (oogenesis, spermatogenesis and embryogenesis), serving to predict a query protein sequence as fertility or non-fertility related protein. The SVM models in the second layer were trained with oogenesis, spermatogenesis and embryogenesis training datasets, separately as binary predictor to further identify the class of the predicted protein in the previous layer (oogenesis, spermatogenesis or embryogenesis).

### Performance evaluation

To quantitatively analyze the efficiency of the proposed predictor, all the SVM models were evaluated with four wildly used performance measures, including specificity (Sp, ability to correctly identify non-fertility), sensitivity (Sn, ability to correctly identify fertility), accuracy (Acc, overall accuracy of the discrimination between positive and negative) and Matthew’s correlation coefficient (MCC, a correlation coefficient between the observed and predicted binary classifications, which takes into account both over- and underpredictions). To make these performance measures easily understood by readers, a set of equations were applied as follows^64^:

1.1 $${\rm{Sn}}=1-\frac{{{\rm{N}}}_{-}^{+}}{{{\rm{N}}}^{+}}\,0\le {\rm{Sn}}\le 1$$

1.2 $${\rm{Sp}}=1-\frac{{{\rm{N}}}_{+}^{-}}{{{\rm{N}}}^{-}}\,0\le {\rm{Sp}}\le 1$$

1.3 $${\rm{Acc}}=1-\frac{{{\rm{N}}}_{-}^{+}+{{\rm{N}}}_{+}^{-}}{{{\rm{N}}}^{+}+{{\rm{N}}}^{-}}\,0\le {\rm{Acc}}\le 1$$

1.4 $${\rm{MCC}}=\frac{1-(\frac{{{\rm{N}}}_{-}^{+}}{{{\rm{N}}}^{+}}+\frac{{{\rm{N}}}_{+}^{-}}{{{\rm{N}}}^{-}})}{\sqrt{(1+\frac{{{\rm{N}}}_{+}^{-}+{{\rm{N}}}_{-}^{+}}{{{\rm{N}}}^{+}})(1+\frac{{{\rm{N}}}_{-}^{+}+{{\rm{N}}}_{+}^{-}}{{{\rm{N}}}^{-}})}}\,-\,1\le {\rm{MCC}}\le 1$$

In the above equations, N^+^ and N^−^ represents the total number of the positive and negative protein sequences investigated, respectively, whereas ${{\rm{N}}}_{-}^{+}$ represents the total number of the positive proteins incorrectly predicted to be negative and ${{\rm{N}}}_{+}^{-}$ represents the total number of false prediction which is incorrectly predicted true. For example, when ${{\rm{N}}}_{-}^{+}=0$ implies that none of the fertility-related proteins were mis-predicted to be a non-fertility related protein, so the sensitivity is 1. Also, when ${{\rm{N}}}_{+}^{-}=0$ implies that none of the non-fertility related proteins correctly predicted as fertility-related proteins, so the specificity is 1.

In the statistical prediction, independent dataset test, subsampling (K-fold cross validation) test, and jackknife cross-validation are often employed to examine the predictive capability of a predictor. As demonstrated in a series of studies^65–68^, among the three test methods, the jackknife cross-validation is deemed as the most objective one that always yield a unique result and hence has been widely used to test the quality of various predictors. However, to save computational time, the five-fold cross-validation (during training the model) and independent test (during testing the model) methods were applied to evaluate the models and generate the performance measurements.

### Feature importance

In machine-learning methods, not all features are equally important for the performance of the trained model, especially for high dimension data. Hence, some features make key contributions and are more important than the others. Thus, it is imperative to employ feature selection technique in order to discover the top ranked feature set according to their predictive contribution. In this study, to discover the most relevant and informative features for discriminating fertility-related proteins, a feature selection method was employed based on a previous study^12^. Running towards this, Rapidminer package (Version 5.2) was applied to fuse 10 different feature weighting algorithms including chi squared statistic, information gain rule, information gain ratio, gini index, deviation, uncertainty, relief, principle component analysis (PCA) and SVM (Supplementary Files 2, S2). Important features were initially defined (based on five general benchmark datasets, a combination of all three fertility-related classes) according to their score from each algorithm (a feature with score >0.5 marked as important). Then, only the features that were marked as important by at least five algorithms were considered. Eventually, common important features in five benchmark datasets were reported as optimal feature set. Furthermore, the optimal feature set was applied to train SVM model, based on five general benchmark datasets, and to investigate the performance of this set for discriminating fertility-related proteins.

## Results and Discussions

The cascade of molecular and intracellular processes which occur in germ cells during spermatogenesis, oogenesis and later embryogenesis are still far from being fully exploited, and this could be a serious bottleneck in the success of fertilization process. Identifying specific fertility related proteins and elucidating their function in regulation of spermatogenesis, oogenesis and embryogenesis are essential for gaining fundamental biological insight intoclinical practice^69^. Machine learning methods, such as SVM, have been used in many fields and their applications in bioinformatics are increasing^12,13,24–27^. However, these methods are yet to be applied in the study of fertility-related proteins and their relevant classes. Therefore, this study aimed to apply SVM-based approach in combination with a comprehensive physical chemical property set to construct a method, which could be used to predict the probability of a sequence, referred to as fertility related protein, as well as its class. The framework diagram of this proposed method is presented in Fig. 1.

### Models performance

To obtain an efficient and robust predictor which can achieve the highest accuracy for identifying fertility-related proteins, it is crucial to apply appropriate training datasets as well as useful physicochemical properties vectors of proteins. Note that no distinct non-fertility related protein dataset exists that can be used here as negative database. Therefore, we took the advantages of our previous method to construct the dataset, which employed a stringency approach to create positive and negative datasets^12,13^. Moreover, comprehensive and informative protein physicochemical properties were applied for fertility-related protein classification, which has been wildly used to predict the different proteins classes^70,71^. A total of 20 training datasets were used for SVM models training, which were associated with oogenesis, spermatogenesis, embryogenesis and general classes (which included all three above mentioned classes). The four performance measurements obtained by training and testing SVM models in different classes along with optimized parameter γ for each dataset are listed in Tables 1–4. The experimental results of an independent assessment of SVM as a binary classifier over different training and testing datasets are stored in the rows in the Table. Using five-fold cross validation approach, SVM model achieved an average prediction Acc of 82.97% with average MCC of 66.95 at the first layer (general class).

**Table 1:** Five-fold cross-validation and Independent evaluation (IE) test results of the SVM method for oogenesis datasets.

Datasets	λ^*^	Five-fold cross-validation test				Independent evaluation test			
		Accuracy (%)	Sensitivity (%)	Specificity (%)	MCC (%)	Accuracy (%)	Sensitivity (%)	Specificity (%)	MCC (%)
1	0.02	82.8	82.86	83.15	65.57	83.33	84.62	80.88	66.7
2	0.001	84.06	83.21	85.04	68.13	83.33	84.62	80.88	66.7
3	0.02	85.33	83.57	86.99	70.71	84.06	86.15	81.16	68.23
4	0.02	82.05	80.71	83.39	63.87	86.23	86.15	84.85	72.4
5	0.001	81.89	81.79	82.37	63.76	82.61	84.62	79.71	65.32
Average	0.01	84	83	85	79.4	84	86	82	67.87

^*^The optimum **λ** parameter value of kernel function of SVM using a grid-search technique based on five-fold cross-validation. Also, the optimum parameter C value was obtained 100 in all of models.

**Table 2:** Five-fold cross-validation and Independent evaluation (IE) test results of the SVM method for spermatogenesis datasets.

Datasets	λ^*^	Five-fold cross-validation test				Independent evaluation test			
		Accuracy (%)	Sensitivity (%)	Specificity (%)	MCC (%)	Accuracy (%)	Sensitivity (%)	Specificity (%)	MCC (%)
1	0.03	82.15	82.12	82.59	64.28	85.99	85.12	85.12	71.88
2	0.03	85.17	81.92	88.02	70.53	84.05	84.3	82.26	68.04
3	0.04	82.73	81.54	83.94	65.47	84.44	84.3	82.93	68.8
4	0.03	83.61	82.88	84.51	67.23	88.33	86.78	88.24	76.56
5	0.05	84.1	81.92	86.06	68.29	81.71	80.99	80.33	63.31
Average	0.04	83.55	82.07	85.02	67.16	84.9	84.29	83.77	69.71

^*^The optimum **λ** parameter value of kernel function of SVM using a grid-search technique based on five-fold cross-validation. Also, the optimum parameter C value was obtained 100 in all of models.

**Table 3:** Five-fold cross-validation and Independent evaluation (IE) test results of the SVM method for embryogenesis datasets.

Datasets	λ^*^	Five-fold cross-validation test				Independent evaluation test			
		Accuracy (%)	Sensitivity (%)	Specificity (%)	MCC (%)	Accuracy (%)	Sensitivity (%)	Specificity (%)	MCC (%)
1	0.02	80.23	80.74	80.38	45.08	80.12	81.41	77.44	47.33
2	0.03	81.05	79.7	82.39	62.14	79.22	77.56	78.06	58.26
3	0.001	80.75	80.59	81.32	65.2	83.43	78.85	84.83	69.42
4	0.03	82.33	81.19	83.54	67.79	78.92	79.49	76.54	62.23
5	0.001	81.43	81.19	82.04	62.85	80.42	82.05	77.58	60.91
Average	0.02	81.15	80.68	81.93	60.61	80.42	79.87	78.89	59.63

^*^The optimum **λ** parameter value of kernel function of SVM using a grid-search technique based on five-fold cross-validation. Also, the optimum parameter C value was obtained 100 in all of models.

**Table 4:** Five-fold cross-validation and Independent evaluation (IE) test results of the SVM method for general datasets.

Datasets	λ^*^	Five-fold cross-validation test				Independent evaluation test			
		Accuracy (%)	Sensitivity (%)	Specificity (%)	MCC (%)	Accuracy (%)	Sensitivity (%)	Specificity (%)	MCC (%)
1	0.001	82.94	84.27	82.48	65.87	81.02	80.41	79.48	64.96
2	0.01	82.56	84.34	81.84	70.1	84.46	83.92	83.19	68.82
3	0.03	82.21	82.85	82.23	64.42	82.26	82.75	80.17	64.48
4	0.04	83.31	83.46	83.63	66.62	82.53	81.29	81.52	64.93
5	0.05	83.87	82.85	84.98	67.76	83.77	80.12	84.57	67.41
Average	0.03	82.97	83.55	83.03	66.95	82.88	81.69	81.78	66.12

^*^The optimum **λ** parameter value of kernel function of SVM using a grid-search technique based on five-fold cross-validation. Also, the optimum parameter C value was obtained 100 in all of models.

At the second layer, the average prediction Acc achieved was 84.00% with average MCC value of 79.40 for oogenesis, an average of 83.55% prediction Acc with average MCC value of 67.16 for spermatogenesis and 81.15% average prediction Acc with an average MCC value of 60.61for embryogenesis (Tables 1–4). Also, the maximum prediction Acc of 85.33%, 83.61%, 82.33% and 82.56% was obtained in oogenesis, spermatogenesis, embryogenesis and general classes, respectively. These results showed that the performance of the classifiers was similar in different classes.

The average Sn and Sp achieved for fertility-related protein prediction was nearly equal and greater than 80% in all classes. This finding indicates that there was no bias in classification, implying an equal chance of identifying the fertility and non-fertility-related proteins correctly (Tables 1–4). To examine the efficiency of the proposed model, five different negative samples were adopted, by random sampling, for each positive dataset. The obtained results on different datasets were slightly different, but consistent in general and the average Acc, Sn, Sp and MCC were all higher than 80% by five-fold cross validation. Therefore, down-sampling of the negative dataset was quite useful.

To further emphasize the effectiveness of the proposed models, roughly 20% of total datasets was retained in each class (testing datasets) for independent evaluation of the final model. Similar results were obtained based on the independent blind test, as the positive datasets could be discriminated from negative datasets with acceptable Acc, MCC, Sn and Sp in all classes. This suggests an encouraging capability and robustness of the proposed method for identifying the fertility-related proteins and their classes (Tables 1–4).

### Feature importance

It is meaningful to determine the most relevant features critical for fertility-related proteins prediction, so that it could be possible to figure out the value of each feature and better understand these proteins. In this study, a stringency feature selection approach was applied on general benchmark datasets to identify optimal feature set. The results led to identification of 22 important features, listed in Table 5, including two amino acid frequency features (isoleucine and serine frequency). Additionally, another important feature group included one dipeptide frequency feature (IA, isoleucine-alanine), 13 features from the CTD descriptor group and five features from the quasi-sequence-order descriptors group and one feature from the conjoint triad descriptor. This finding was consistent with a previous study, which confirmed serine as an important feature for oogenesis-related proteins prediction^12^. Interestingly, the importance of serine in fertility-related proteins, especially oogenesis^72–74^, was also highlighted in previous studies^75–80^. Also, how members of the TGF-β superfamily induce the constitution of hetero-oligomeric complexes of two distantly related types of serine/threonine kinases was also highlighted^12^.

**Table 5:** The top 22 important features selected by attribute weighting feature selection method for general dataset.

order	Descriptor	Protein feature	Feature group
1	**S**	Serine	Amino Acid Composition
2	I	Isoleucine	Amino Acid Composition
3	IA	Dipeptide Composition (Isoleucine-Alanine)	Amino Acid Composition
4	solventaccess.Group1	Solvent Accessibility attribute of Composition	CTD
5	solventaccess.Group3	Solvent Accessibility attribute of Composition	CTD
6	Schneider.Xr.S	QSO in QSOD using Schneider-Wrede distance	Quasi-sequence-order
7	Grantham.Xr.I	QSO in QSOD using normalized Grantham chemical distance	Quasi-sequence-order
8	Grantham.Xd.1	QSO in QSOD using normalized Grantham chemical distance	Quasi-sequence-order
9	prop7.Tr2332	Solvent Accessibility attribute of Transition	CTD
10	prop5.G2.residue0	Charge attribute of Distribution	CTD
11	prop5.G2.residue25	Charge attribute of Distribution	CTD
12	prop5.G2.residue50	Charge attribute of Distribution	CTD
13	prop5.G2.residue75	Charge attribute of Distribution	CTD
14	prop5.G2.residue100	Charge attribute of Distribution	CTD
15	VS333	Conjoint Triad	Conjoint Triad
16	prop2.G1.residue0	Normalized van der Waals Volume attribute of Distribution	CTD
17	prop2.G1.residue25	Normalized van der Waals Volume attribute of Distribution	CTD
18	prop2.G1.residue50	Normalized van der Waals Volume attribute of Distribution	CTD
19	prop2.G1.residue75	Normalized van der Waals Volume attribute of Distribution	CTD
20	prop2.G1.residue100	Normalized van der Waals Volume attribute of Distribution	CTD
21	Schneider.Xr.I	QSO in QSOD using Schneider-Wrede distance	Quasi-sequence-order
22	Grantham.Xr.S	QSO in QSOD using normalized Grantham chemical distance	Quasi-sequence-order

In order to provide more evidence regarding the important role of serine in spermatogenesis, first the role of protamine in maintaining spermatogenesis and spermatozoa quality was highlighted. Protamine is well known to function as an essential protein for sperm nuclear condensation. It is a very simple and specialized protein which comprises 44 amino acid residues that belongs to three amino acid types: arginine, glycine, and serine. In all vertebrate, two structural elements have been identified in protamines. The first, which is facilitating binding of protein to DNA, is a series of small ‘anchoring’ domains containing multiple arginine or lysine amino acids. The second one is a multiple residues of serine and threonine, which could potentially act as phosphorylation sites^81^. There is also enough evidence that phosphorylation-dephosphorylation events control the deposition of protamines on sperm chromatin and the subsequent chromatin condensation. Protamines are highly phosphorylated, shortly after their synthesis and before binding to DNA, whereas they become largely dephosphorylated during sperm maturation^82^. Sperm function is regulated by the activation of intracellular signaling systems during fertilization, which control protein phosphorylation. Protein phosphorylation is involved in modification of proteins, post-translationally, that allows the control of various cellular processes by the cell. Phosphorylation mostly occurs on serine or threonine residues, but it is also encountered on tyrosine residues. Protein kinases and phosphatases are enriched in sperm and regulate the phosphorylation state of phosphoproteins. Serine/threonine phosphorylation are known to occur in spermatozoa and has a pivotal role in the regulation of sperm motility^83^. As a consequence of improved understanding about serine amino acid, perturbation of the cell cycle at the G1–S and/or G2–M transitions is likely to occur just following serine deficiency. It was recently demonstrated that in the absence of L-serine, the fibroblast cells prepared from knockout embryos are unable to proliferate. Therefore, the serine synthesized within cells of embryos plays a crucial role in cell cycle progression of a variety of cell types including radial glia during fetal development. With regard to cell cycle dysregulation in the knockout spinal cord, it is notable that radial glia cells in the ventricular zone expresses cell proliferation markers PCNA and Ki67, and are not assumed to enter neuronal differentiation or a mitotically quiescent G0-like state^84^.

Isoleucine is believed to be genetically related to male fertility through its synthetic and metabolic activities^85^. For instance, mutation of encoding gene of ubiquitin-specific protease 26 (responsible for a valine to isoleucine change) has been reported to cause male infertility and adversely affect the testicular function^86^. Cytochrome P4501A1 participates in isoleucine–valine exchange; mutation of its heme-binding region is also associated with infertile men^87^. Haqq *et al*., highlighted the importance of isoleucine in sex-determining region Y (SRY) protein, specifically the orientation of isoleucine side chain in DNA minor groove^88^. Isoleucine plays essential roles in embryogenesis, particularly during fetal development^89^. Isoleucine is among the branched chain amino acids (BCAA), which have been considered as one of the vital elements in fetus development. In this regard, it has been shown that BCAA supplemented diets can improve the gene and protein expression of IGF-1 and IGF-2 in fetal liver, consequently leading to amelioration of fetal growth restrictions^90^.

A SVM-based machine learning method was also built using the general datasets for the prediction of fertility-related proteins based on optimal feature set. Similarly, five-fold cross validation and independent tests were applied to estimate the performance. The performances of the trained model using optimal feature set on general datasets are shown in Table 6. When optimal feature set were used, the model could reach (on average) 81.68%, 79.44%, 79.98% and 59.9 (evaluated by five-fold cross validation) for Sn, Sp, Acc and MCC, respectively. Also, the average Sn, Sp, Acc and MCC were 78.53%, 76.15%, 78.32% and 55 based on independent test, respectively (Table 6). As shown in Table 6, once optimal feature set was used for training the SVM model compared to the original feature set, there was decrease in the Acc by 3% and 4.6% by five-fold cross validation and independent test, respectively. This can be somehow attributed to the stringent feature selection method, which led to finding only 22 very important features. However, the models trained by SVM using the optimal feature set could classify fertility-related proteins and their classes, with a relatively high accuracy as well as with a relatively high and equal sensitivity and specificity (Table 6). Overall, the results in this study imply that a comprehensive feature set is more efficient than selected features in recognizing fertility-related protein. It is in complete agreement with previous studies suggesting that using a comprehensive and proper protein feature set gives the better result^12,13,91^.

**Table 6:** Five-fold cross-validation and independent evaluation test results of the SVM method for general datasets with selected features.

Datasets	λ^*^	Five-fold cross-validation test				Independent evaluation test			
		Accuracy (%)	Sensitivity (%)	Specificity (%)	MCC (%)	Accuracy (%)	Sensitivity (%)	Specificity (%)	MCC (%)
1	0.05	79.95	82.1	79.15	59.9	79.5	80.12	77.18	58.99
2	0.08	79.74	80.88	79.53	59.46	77.99	77.78	76	55.9
3	0.09	80.12	81.29	79.88	60.22	77.03	78.36	74.24	54.11
4	0.04	79.91	82.24	79.02	59.68	77.58	78.07	75.21	55.13
5	0.09	80.19	81.9	79.63	60.37	79.5	78.36	78.13	50.91
Average	0.07	79.98	81.68	79.44	59.92	78.32	78.53	76.15	55

^*^The optimum **λ** parameter value of kernel function of SVM using a grid-search technique based on five-fold cross-validation. Also, the optimum parameter C value was obtained 100 in all of models.

### Software development

A software support is required to make the development of new classification models publicly available. In order to enhance the value of our evolving software into practical applications, a two-layer classifier called PrESOgenesis (Predict Embryo-, Spermato- and Oogenesis) has been provided freely at https://github.com/mrb20045/PrESOgenesis. The best model in each class was selected based on five-fold cross validation results for the development of the predictor. The first layer predicts the input sequence, whether it is fertility-related or not, using a binary SVM classifier. If not, the classifier is automatically stopped. If yes, the sequence is considered as a fertility-related protein candidate and is subsequently submitted into the second layer. Then, in this layer, three binary SVM classifiers (for oogenesis, spermatogenesis, and embryogenesis classes) are applied to determine to which classes of fertility-related proteins they are assigned. The class is designated as one of the three categories (oogenesis, spermatogenesis or embryogenesis), on the basis of highest SVM score. In this study, a two-layer classifier was proposed for predicting fertility-related protein. The high efficiency of this method has been reported in previous studies such as predicting membrane proteins^92^, enhancer prediction^93^, remote protein homology detection^94^, identifying piwi-interacting RNAs^34^ and miRNA Drosha processing site detection^95^.

PrESOgenesis can be used by a wide variety of researchers with limited knowledge of the SVM computing environment, since it just requires simply upload sequence(s) in FASTA format for prediction. The user receives the prediction reports as output and the estimated probability scores. Probability score (ranging from 0 to 1), which reflect the confidence of decisions, is assigned to each predicted protein. PrESOgenesis marks inputted sequences with probability score >0.5 as fertility-related protein (first layer) or one of the fertility-related classes (second layer). However, the threshold can be adjusted by users to adjust the false positive results (higher score can lead to lower false positive).

Protein or mRNA transcript sequences can be used as input sequences to PrESOgenesis. Accordingly, the software was equipped with TransDecoder tool (version 3.0.1, http://transdecoder.sourceforge.net), which obtained the candidate protein region based on the open reading frame (ORF) and nucleotide composition. Then, the predicted protein sequences were automatically inputted to the first layer of classifier for predicting their potential as fertility-related proteins. This capability can be applied to annotate the unknown transcripts that have been generated from deep sequencing projects such as RNA-Seq studies.

In this study, to address the issue of whether the new PrESOgenesis has a better or at least comparable performance to the previously introduced OOgenesis_Pred, a comparison of the two softwares was made. Towards a fair performance comparison, a negative sample including 1000 protein sequences were randomly selected from the negative dataset and were used as query. To avoid bias, none of the sequences in the negative sample appeared in the datasets used to train both of software. The results showed that PrESOgenesis achieved better performance in predicting the protein sequences as non-oogenesis related protein than OOgenesis_Pred. Using these two software, 140 (by PrESOgenesis) and 184 (by OOgenesis_Pred) proteins as non-oogenesis-related proteins were identified (Supplementary File 3). The higher performance of PrESOgenesis can be attributed solely with certainty the two-layer prediction architecture of this software, which is equivalent to making full use of the interclass relationships between fertility and non-fertility related proteins. Since PrESOgenesis is the first classifier ever developed for identifying fertility related proteins, it is not possible to compare its accuracy precisely against its counterparts for exactly the same purpose. However, its power can be compared with some related tools in other areas.

The trained models in this study could achieve an accuracy more than 81%^8,11–16,96^. The accuracy and robustness of the model could also be evaluated using new fertility-related proteins belonging to different classes which were added into Uniprot databases (release 2017_10) since after PrESOgenesis had been developed. Therefore, to test the prediction power of PrESOgenesis, proteins sequences were collected again by searching the UniProtKB database (release 2017_10) with gene ontology terms “oogenesis”, “spermatogenesis” and “embryogenesis” and new added protein sequences were retrieved from the datasets. 18, 39 and 144 new protein sequences were obtained in oogenesis, spermatogenesis and embryogenesis classes, respectively. Interestingly, PrESOgenesis could properly predict 15 of 18 (83.33%), 35 of 39 (89.74%) and 117 of 144 (81.25%) sequences. These results further proved the reliability of PrESOgenesis for identifying fertility-related proteins (Supplementary File 4).

### Limitation and future work

Three classes of fertility-related proteins have been focused on in this study; though there are other relevant protein classes to fertility. Incorporating such proteins data and thus complementing the training datasets, may well improve the accuracy of predictors and help to reduce the false positive rates. Therefore, in future work it is necessary to attempt to add other fertility-related protein classes to the training datasets, which can be used in combination to further improve the reliability of the predictor.

Since both user-friendly and publicly accessible web-servers^97–99^ and databases^100,101^ represent the direction of developing new prediction method, efforts shall be made in future work to provide a web-server for the prediction method presented in this paper.

## Conclusions

With the advent of post-genomic era and increasing use of computational techniques, the computational annotation of proteins has become a priority research area nowadays. In this study, the hypothesis was that fertility-related proteins possess some characteristics which distinguish their sequences from their non-fertility counterpart proteins. To this end, six sequence-based feature descriptors were integrated with a vector of 1,920 dimensions to facilitate the analysis and identification of fertility-related proteins and their classes. Here, for the first time, a two-layer classification framework was developed based on the SVM method, called PrESOgenesis. At the first layer, each protein was classified by SVM classifier to determine whether it is a fertility-related protein or not. If so, it was further classified by three SVM models to determine to which functional classes it belongs. Five-fold cross-validations along with independent test indicated that the proposed method is very powerful and promising. Also, an in-depth feature analysis was used to identify the most important features for identifying fertility-related proteins. A total of 22 important features were identified such as serine and isoleucine frequency and showed that they significantly contribute to the prediction. It is anticipated that PrESOgenesis will become a very useful bioinformatics tool for predicting fertility-related proteins.

## Electronic supplementary material

Supplementary File 1

Supplementary File 2

Supplementary File 3

Supplementary File 4
