# Supplementary material for: PrESOgenesis: A two-layer multi-label predictor for identifying fertility-related proteins using support vector machine and pseudo amino acid composition approach
Source: Sci Rep. 2018 Jun 13;8:9025. doi: 10.1038/s41598-018-27338-9 (PMC5998058; doi:10.1038/s41598-018-27338-9)
Supplement: Supplementary file 2 [file 41598_2018_27338_MOESM2_ESM.pdf]

PrESOgenesis: A two-layer multi-label predictor for identifying fertility-related proteins using support vector machine and pseudo amino acid composition approach

Mohammad Reza Bakhtiarizadeh[a*], Maryam Rahimi[a], Abdollah Mohammadi-Sangcheshmeh[a], Vahid Shariati J[b], Seyed Alireza Salami[c**]

[a] Department of Animal and Poultry Science, College of Aburaihan, University of Tehran, Tehran, Iran

[b] Plant Molecular Biotechnology Department, National Institute of Genetic Engineering and Biotechnology, Tehran, Iran

[c] University of Tehran, Tehran, Iran

Supplementary File 2. Description of applied structural and physicochemical protein features along with description of applied 10 different algorithms of attribute weightings in this study.

S1. Description of applied structural and physicochemical protein features in this study.

Amino acid composition (AAC)

AAC is the fraction of each amino acid type within a protein. The fractions of all 20 natural amino acids are defined as [1]:

$$F(r) = \frac{Number\ of\ amino\ acid\ of\ type\ r}{Length\ of\ amino\ acid\ sequence} \qquad r = 1, 2, \ldots, 20.$$

Dipeptide Composition (DC)

Dipeptide composition of a protein is employed to convert a variable length protein sequence to a fixed 400 feature vectors. An amino acid composition offers only information of sequence but

disregard the sequence order information. As mentioned above the DC offers 400 descriptors, calculated as [2]:

$$F\,(r, s) = \frac{\text{number of dipeptide represented by amino acid type } r \text{ and type } s}{\text{total number of all possible dipeptide}} \qquad r, s = 1, 2, \ldots, 20$$

Autocorrelation features

Autocorrelation features are based on the distribution of amino acid properties along the sequence. In this study, we used eight amino acid properties to evaluate these features including normalized average hydrophobicity scales, average flexibility indices, polarizability parameter, free energy of solution in water, residue accessible surface area in tripeptide, steric parameter, relative mutability and residue volume. Three types of autocorrelation features are defined here and described below.

Normalized Moreau-Broto autocorrelation descriptors can be computed as:

$$AC(D) = \sum_{i=1}^{N-d} (p_i p_{i+d}) \qquad d = 1, 2, \ldots, \text{nlag}$$

Moran autocorrelation descriptors application to protein sequence can be calculated as:

$$I(d) = \frac{\frac{1}{N-d}\sum_{i=1}^{N-d} (p_i - \acute{\bar{p}})\,(p_{i+d} - \acute{\bar{p}})}{\frac{1}{N}\sum_{i=1}^{N} (p_i - \acute{\bar{p}})^2} \qquad \text{d} = 1, 2, \ldots, 30$$

Where d and $p_i$and p_{i+d} are defined properties of amino acid at the position i and i + l, respectively. and $\acute{\bar{p}}$ is the considered property P along the sequence, i.e.,

$$\acute{\bar{p}} = \frac{\sum_{i=1}^{N} p_i}{\text{N}}$$

Geary autocorrelation descriptors for protein sequence can be calculated as:

$$C(d) = \frac{\frac{1}{2(N-d)} \sum_{i=1}^{N-d} (p_i - p_{i+d})^2}{\frac{1}{N-1} \sum_{i=1}^{N} (p_i - \acute{p})^2} \qquad d = 1, 2, ..., 30$$

Hence in this study, 8*30 = 240 of each correlation attributes with total of 240*3 = 720 attributes were defined [3].

Composition, transition and distribution features (CTD)

These descriptors are proposed and described by Dubchak et al. (1995) and Dubchak et al. (1999). To compute these descriptors, the amino acids of a protein sequence are divided in three classes due to its attribute: neutral, polar and hydrophobicity by using properties of physiochemical include hydrophobicity, normal Vander Waals volume, polarity, polarizability, charge secondary structure, and solvent accessibility and then each amino acid is represented by one of the indices 1, 2, 3 according to which class it belonged. Three descriptors, Composition (C), Transition (T), and Distribution (D) were defined for a given attribute by using:

Composition:

$$C = \frac{\text{the number of amino acid type } r \text{ in the encoded sequence}}{\text{the length of the sequence}} \qquad r = 1, 2, 3$$

Transition:

$$T_{rs} = \frac{\text{the numbers of dipeptide encoded as``rs''and``sr''}}{\text{the length of the sequence} - 1} \qquad rs = \text{`12', `13', `23'}$$

The distribution descriptor represents the distribution of every property in the sequence. It calculates five descriptors of distribution for every attribute in first residue, 25% residue, 50% residue, 75% residue, and 100% residue, respectively, for each specified encoded group. Therefore in this study, total 7*3 = 21 composition attributes, 7*3 = 21 transition attributes, and 7*3*5 = 105 distribution attributes were defined [1].

Conjoint Triad descriptors

Conjoint triad descriptors are developed by Shen et al. (2007). These conjoint triad descriptors summarize the attributes of protein pairs according to the amino acid classification. In this way, every sequence of protein is defined by a vector space consisting of amino acids descriptors. The 20 amino acids were clustered into various groups due to their volumes of the side chains and dipoles to decrease vector space dimensions. The conjoint triad descriptors are computed as follow:

$$d_i = \frac{\mathrm{f}_i - \min\{f_1, f_2, \dots, f_{343}\}}{\max\{f_1, f_2, \dots, f_{343}\}}$$

V is the vector space of the sequence features; each feature (V_i) represents a triad composed of three consecutive amino acids; F is the frequency vector corresponding to V, and the value of the i-th dimension of F(f_i) is the frequency that vi-triad appeared in the protein sequence [2].

Quasi-sequence-order descriptors

Sequence-order descriptors are developed by Chou (2000). They are calculated from the distance matrix between the 20 amino acids pair.

Sequence-order-coupling number

The d-th rank sequence-order-coupling number is calculated as:

$$T_d = \sum_{i=1}^{N-d} \left(d_{i,i+d}\right)^2 \qquad \text{d = 1, 2, . . . , maxlag}$$

Where $d_{i,i+d}$is the distance between the two amino acids at position *i* and *i* + *d*. two physiochemical distance matrix such as Schneider, Wrede and Grantham where maxlag(d) = 30. So the total (d*2 = 60) number of features of sequence order coupling numbers are extracted.

Quasi-sequence-order descriptors

For each amino acid type, a quasi-sequence order descriptor can be calculated as:

$$x_r = \frac{\mathrm{f}_r}{\sum_{r=1}^{20} f_r + \mathrm{w} \sum_{d=1}^{maxlag} T_d} \qquad \mathrm{r} = 1, 2, \ldots, 20$$

$$x_d = \frac{\mathrm{WT}_{d-20}}{\sum_{r=1}^{20} f_r + \mathrm{w} \sum_{d=1}^{maxlag} T_d} \qquad \mathrm{d} = 21, 22, \ldots, 20+ \text{ maxlag}$$

Here, f_r is the normalized occurrence of amino acid of type r and w is the weighting factor. Here, two physiochemical distance matrix such as Schneider, Wrede and Grantham where maxlag(d) = 30. So 50*2 = 100 features of quasi-sequence order features are extracted [4].

Pseudo amino acid composition (PAAC)

Descriptors of pseudo amino acid composition are proposed in Chou (2001). Recently various powerful open access soft-wares, called 'PseAAC-Builder' (Du et al., 2012), 'propy' (Cao et al., 2013), and 'PseAAC-General' (Du et al., 2014), were developed to generate various descriptors of pseudo amino acid composition. PAAC descriptors are called the type 1 pseudo-amino acid composition are computed by using the three properties: the original hydrophobicity values $H_1^0(i)$, the original hydrophilicity values $H_2^0(i)$ and the original side chain masses $M^0(i)$ of the 20 amino acids, respectively. The correlation function between these three properties can be calculated as:

$$\theta(R_i, R_i) = \frac{1}{3}\left\{\left[H_1(R_i) - H_1(R_j)\right]^2 + \left[H_2(R_i) - H_2(R_j)\right]^2 + \left[M(R_i) - M(R_j)\right]^2\right\}$$

By using these correlation values, a set of sequence order correlated features are defined as

$$\theta_\lambda = \frac{1}{N-\lambda} \sum_{i=1}^{N-\lambda} \theta(R_i, R_{i+\lambda}) \qquad \lambda = 1 \; to \; 30$$

f_n can be frequency of the normalized occurrence of the 20 amino acid in the sequence of protein, a set of 20 + λ pseudo amino acid composition descriptors can be defined as:

$$PAAC_n = \frac{f_n}{\sum_{r=1}^{20} f_r + w \sum_{j=1}^{30} \theta_j} \qquad 1 \leq n \leq 20 \text{ and } \ w = 0.1$$

$$PAAC_n = \frac{\theta_{n-20}}{\sum_{r=1}^{20} f_r + w \sum_{j=1}^{30} \theta_j} \qquad 21 \leq n \leq 50 \text{ and } \ w = 0.1$$

Amphiphilic pseudo-amino acid composition (APAAC) was developed by Chou (2001). APAAC is the type 2 pseudo-amino acid composition. The descriptors of APAAC are similar to the PAAC descriptors. in these qualities, sequence order factors can be computed as:

$$\tau_{2\lambda} = \frac{1}{N-\lambda} \sum_{i=1}^{N-\lambda} H_{i,i+\lambda}^2 \qquad \lambda = 1 \text{ to } 30$$

So, a set of descriptors of Amphiphilic pseudo-amino acid composition (APAAC) are calculated as:

$$P_c = \frac{f_c}{\sum_{r=1}^{20} f_r + w \sum_{j=1}^{2\lambda} \tau_j} \qquad 1 < c < 20 \text{ and } \ w = 0.1$$

$$PAAC_n = \frac{w\tau_u}{\sum_{r=1}^{20} f_r + w \sum_{j=1}^{2\lambda} \tau_j} \qquad 21 < u < 20 + 2\lambda \text{ and } \ w = 0.1$$

Then total 50 pseudo amino acid composition features and 80 Amphiphilic pseudo-amino acid composition features were calculated [5, 6].

S2. Description of applied 10 different algorithms of attribute weightings in this study [7].

Weight by information gain: This operator computed the relevance of a feature by calculating the information gain in class distribution.

Weight by rule: This operator computed the relevance of a feature by calculating the error rate of a One R Model on the example set without this feature.

Weight by information gain ratio: This operator computed the relevance of a feature by calculating the information gain ratio for the class distribution.

Weight by chi squared statistic: This operator computed the relevance of a feature by calculating, for each attribute of the input example set, the value of the chi-squared statistic with respect to the class attribute.

Weight deviation: By the standard deviations of all attributes, this operator formed weights. The values were normalized through the minimum, the average or the maximum of the attribute.

Weight by Gini index: This operator computed the relevance of an attribute by calculating the Gini index of the class distribution, if the given example set would have been split according to the feature.

Weight by relief: This operator calculated the relevance of features by sampling examples and comparing the value of the current feature for the nearest example of the same and of a various class. This version also worked for multiple classes and regression data sets. The resulting weights were normalized into the interval between 0 and 1.

Weight by uncertainty: This operator calculated the relevance of an attribute by measuring the symmetrical uncertainty with respect to the class.

Weight by SVM (Support Vector Machine): This operator calculated feature weights by the coefficients of the normal vector of a linear SVM.

Weight by PCA (Principle Component Analysis): This operator calculated feature weights by the first of the essential constituent.

References

1. Yuan, Y., et al., *Prediction of interactiveness of proteins and nucleic acids based on feature selections.* Molecular diversity, 2010. **14**(4): p. 627-633.
2. Xiao, N., Q.-S. Xu, and D.-S. Cao, *protr: R package for generating various numerical representation schemes of protein sequences.* 2017.
3. Li, Z.-C., et al., *Prediction of protein structure class by coupling improved genetic algorithm and support vector machine.* Amino acids, 2008. **35**(3): p. 581-590.
4. Li, Z.-R., et al., *PROFEAT: a web server for computing structural and physicochemical features of proteins and peptides from amino acid sequence.* Nucleic Acids Research, 2006. **34**(suppl_2): p. W32-W37.
5. Shen, H.-B. and K.-C. Chou, *PseAAC: a flexible web server for generating various kinds of protein pseudo amino acid composition.* Analytical biochemistry, 2008. **373**(2): p. 386-388.
6. Chou, K.-C., *Using amphiphilic pseudo amino acid composition to predict enzyme subfamily classes.* Bioinformatics, 2004. **21**(1): p. 10-19.
7. Ebrahimi, M., et al., Prediction of thermostability from amino acid attributes by combination of clustering with attribute weighting: a new vista in engineering enzymes. PloS one, 2011. **6**(8): p. e23146.
